# Factors Influencing Women’s Attendance to Postnatal Clinics in the Primary Healthcare Centers in the Kingdom of Bahrain, 2023

**DOI:** 10.7759/cureus.55834

**Published:** 2024-03-09

**Authors:** Fatema Tawfeeq, Maryam Alkhaldi, Zahra AlAwainati, Fatema Mansoor, Hajer AlShomeli, Basheer Makarem, Hala AlAsomi

**Affiliations:** 1 Department of Family and Community Medicine, College of Medicine and Medical Sciences, Arabian Gulf University, Manama, BHR; 2 Department of Mother and Child Services, Primary Health Care Center, Ministry of Health, Manama, BHR

**Keywords:** postnatal, mothers, determinants, utilization, postpartum

## Abstract

Background: Despite the significance of postnatal care for maternal health, the attendance rate of mothers at postnatal clinics (PNCs) in primary healthcare (PHC) centers in Bahrain is low. This study aims to identify factors influencing women's attendance at PNC in PHC centers in the Kingdom of Bahrain and to propose strategies for service improvement.

Methods: In January 2023, we conducted a cross-sectional study. We selected mothers who gave birth between six and 12 months before the survey and met inclusion criteria using systematic simple random sampling and obtained consent (n = 319). Data collection utilized an electronic, structured, interviewer-administered questionnaire, and analysis was carried out using SPSS version 28 (IBM Corp., Armonk, NY).

Results: Out of the 319 participants, 31% were unaware of the existence of PNC, 42% were aware but did not attend, and 27% were aware and had attended the clinic. Mother and child department clerks, doctors, health education boards, and midwives served as the primary sources of information about the PNC for only 34%, 16%, 16%, and 11% of participants, respectively. Understanding the importance of PNC, the services offered by a healthcare worker, the booking process, having a vaginal delivery, and prior experience with a PNC visit were significantly associated with PNC attendance (p = 0.0046, p = 0.027, p < 0.001, p = 0.028, and p < 0.001, respectively). The attendance of 81 mothers, representing 94% of the total women who attended the clinic, was driven by their acknowledgment of the service's importance. Childcare responsibilities, perception of the visit as unimportant, and reluctance to undergo a pelvic examination were the top reasons for not attending the clinic, despite 41%, 38%, and 37% of participants being aware of it.

Conclusion: Postnatal care attendance remains suboptimal for mothers in Bahrain. Awareness of the clinic's presence, counseling by healthcare providers, and prior experience with PNC visits were found to be significant determinants of attendance.

## Introduction

The postnatal period is a critical phase in ensuring the health and survival of mothers, as it is a period when various complications can arise [1]. Postnatal care plays a pivotal role in reducing maternal morbidity and mortality by encompassing a comprehensive evaluation of the mother's health, including the monitoring of perinatal complications, such as gestational diabetes [2-4], pregnancy-induced hypertension [5], and anemia [6,7]. Additionally, it offers preventive measures such as screening for postpartum depression [8-10], cervical cancer [11], domestic violence [12,13], and immunization [14]. Postnatal care also includes counseling on essential topics, such as breastfeeding [15-17], family planning [18-20], sexuality [21,22], exercise [23,24], and nutrition [25,26]. Dedicated postnatal care services offer specialized attention and support tailored to the distinct challenges and health requirements during the postpartum period. Despite its significance, the utilization of postnatal care services remains low, as evidenced by several studies [27-32]. These studies have identified various factors contributing to low attendance, including patient-related factors [33-39], facility-related factors [38,40,41], cultural beliefs [41], and factors associated with healthcare workers [42,43].

Notably, there exists a significant disparity in recommendations regarding the timing of postnatal visits [44]. The World Health Organization (WHO) guidelines currently advise that women should receive postnatal care within 24 hours after childbirth, with at least three subsequent visits at 48-72 hours, seven to 14 days, and six weeks postpartum [1]. In contrast, the National Institute for Health and Care Excellence (NICE) recommends that all women and their infants should receive postnatal care within the first eight weeks following childbirth [45].

Bahrain, as a nation, has established its own guidelines for postnatal care in primary healthcare (PHC) settings. In Bahrain, the postnatal visit consists of a single appointment that can be scheduled between six weeks and six months after delivery [46]. This visit serves the purpose of conducting a comprehensive assessment of the mother's physical and mental health, as well as delivering preventive services and counseling on various vital postpartum issues. Despite being important, accessible, and cost-free, the annual statistics from the Central Informatics Organization of Bahrain's 2019 internal audit revealed that only 29% of those who had attended antenatal clinics in PHC centers in Bahrain had availed themselves of postnatal care services at PHC centers. This low attendance rate warrants attention, especially in light of the fact that postnatal services were delivered through teleconsultation during the COVID-19 pandemic in 2020 and 2021. Furthermore, despite the prolonged low attendance rate, the underlying factors have not been comprehensively studied in Bahrain. Therefore, this study was initiated to explore the determinants of postnatal maternity service utilization and to identify the factors that influence women's attendance at postnatal clinics (PNCs) in Bahrain.

## Materials and methods

Study design and participants

This cross-sectional study included 319 participants, comprising Bahraini women and non-Bahraini women married to Bahraini nationals who had given birth within the range of six to 12 months before the commencement of the study. Participants were recruited during their attendance at child screening visits for infants at the ages of six, nine, and 12 months. The sample size was determined utilizing a simple random sampling formula: n = [Za/2^2 * P * (1-P)] / E^2, where Za/2 corresponds to the standard variable at the (1-a)% confidence level (with a value of 1.96 for a 95% confidence interval), P signifies the proportion of new mothers who attended PNCs in the PHC centers in Bahrain, estimated at 29% based on 2019 statistics, and E represents the margin of error set at 5%; the calculated sample size is 317. To ensure that the income factor did not influence the decision to attend the postnatal visit, non-Bahraini women not married to Bahrainis were excluded, as postnatal services are provided exclusively free of charge to Bahraini women and non-Bahraini women who are married to Bahraini men.

Data collection

Data collection occurred from January 2 to January 17, 2023. We employed a lottery-based method to randomly select five health centers, one from each health region. The sample size was allocated among the selected health centers in proportion to the rate of attendance at the child screening clinics in those centers. Every third woman who met the inclusion criteria and provided consent was interviewed using an interviewer-administered electronic structured questionnaire.

Data analysis

The collected data were analyzed using the Statistical Package for Social Sciences (SPSS) version 28 (IBM Corp., Armonk, NY). Categorical variables were expressed as frequencies and percentages, while continuous variables were presented as means and standard deviations. Bar graphs and clustered bar charts were employed to illustrate the frequency or percentage of categorical (qualitative) variables. The chi-square test was used to assess associations between categorical variables, including socio-demographic characteristics, obstetric characteristics, and awareness and utilization of maternal healthcare services. A p-value of less than 0.05 was considered statistically significant.

Ethical considerations

Ethical approval was obtained from the Research and Ethics Committee at the College of Medicine and Medical Sciences, Arabian Gulf University, as well as the Research and Ethics Committees at Primary Healthcare in the Kingdom of Bahrain. Informed written consent was obtained from all participants by the interviewers before data collection. The participants were informed about the study's purpose, and their anonymity was assured. The interviews were conducted in a private and confidential manner, and data confidentiality was strictly maintained throughout the study.

## Results

A total of 319 mothers who brought their children to the child screening clinic in PHC centers in Bahrain between the ages of six and 12 months between the 2nd and 17th of January 2023 were consented and interviewed in this study. The sociodemographic and obstetric characteristics of mothers are shown in Table 1.

**Table 1:** Sociodemographic and obstetric characteristics of the participants (n = 319).

Variable	n (%)
Age (mean ± SD = 29.77 ± 5.407)	
<25 years	54 (17)
25-34 years	202 (63.3)
≥35 years	63 (19.7)
Level of education	
Secondary school or below	144 (45.1)
College and above	175 (54.9)
Employment status	
Unemployed	220 (69)
Employee or student	99 (31)
Marital status	
Married	318 (99.7)
Divorced	1 (0.3)
Husband's level of education (n = 318)	
Secondary school or below	197 (61.9)
College and above	121 (38.1)
Number of deliveries	
1	107 (33.5)
2-3	153 (48)
4 or more	59 (18.5)
Mode of delivery	
Vaginal delivery	200 (62.7)
Cesarean section	119 (37.3)
Intention of pregnancy	
Planned	197 (61.8)
Unplanned	122 (38.2)
Acceptance of unplanned pregnancy (n = 122)	
No	8 (6.6)
Yes	114 (93.4)
Attendance of antenatal clinic visits during the last pregnancy	
Some visits	21 (6.6)
All visits	298 (93.4)

**Figure 1:**
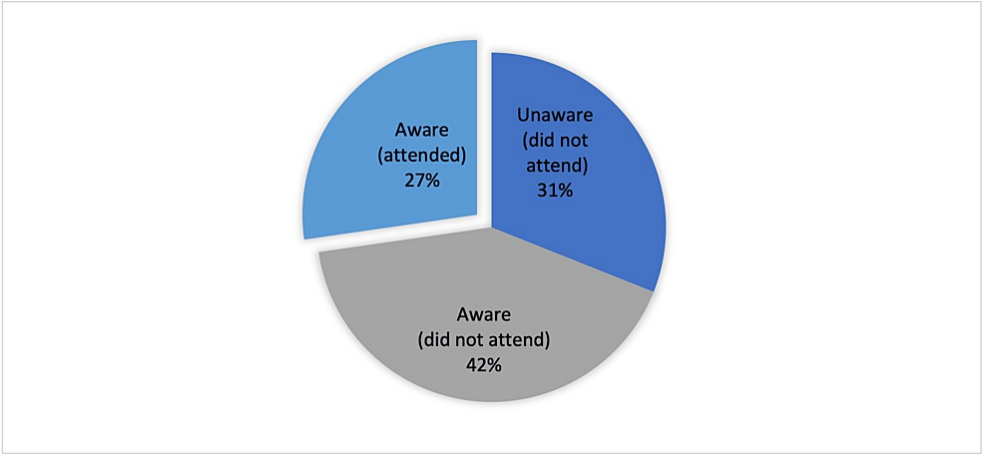
Attendance and awareness rate of postnatal clinics in primary healthcare centers among participants (n = 319).

**Figure 2:**
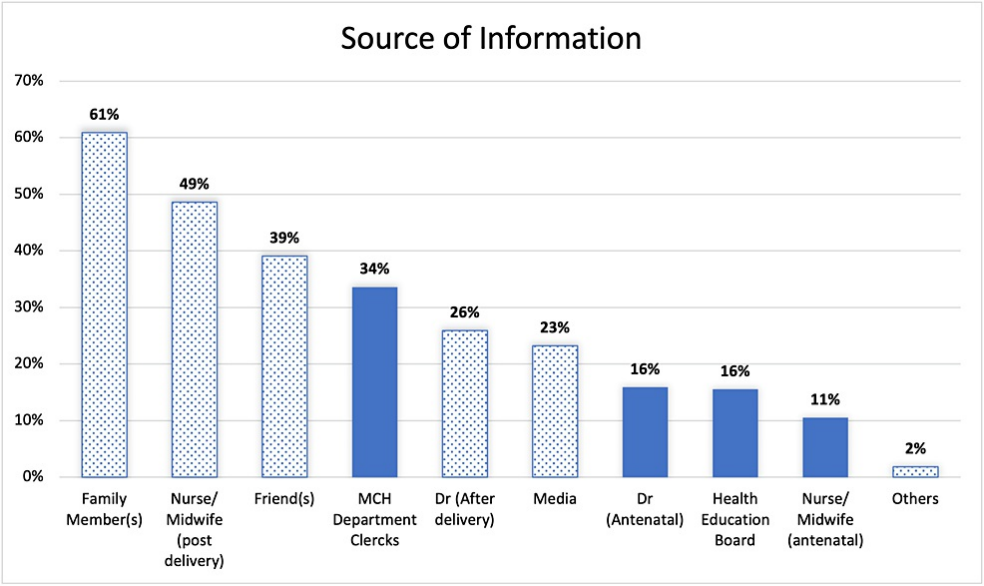
Source(s) of information about the presence of postnatal clinics in primary healthcare centers among study participants who were aware of the service (n = 220; multi-response question). MCH: mother and child health.

Out of 319 participants, only 87 (27%) attended the PNC after the last delivery. A total of 99 (31%) mothers reported that they were unaware of the presence of PNC in PHC centers in Bahrain, and thus they did not attend. While 133 (42%) participants were aware but did not attend the clinic (Figure 1). Mothers who were aware of the clinic (n = 220) were asked to choose, among a list of sources, where they got the information about the clinic (Figure 2).

Among 220 mothers who were aware of the PNC in PHC centers in Bahrain, 146 (66%) were informed about it by healthcare providers (doctors and/or midwives in antenatal visits or post-delivery).

Of those, only 50 (34%) participants were counseled about the importance of PNC by their healthcare providers while the remaining were not. Fifty-seven participants (59%) of those who did not receive counseling about the importance of postnatal care did not attend the clinic after the last delivery. On the other side, 29 (58%) mothers who received counseling about the importance of postnatal visits attended the clinic (p = 0.046).

Out of 146 participants who were aware of the presence of PNC in PHC, 76% (111 participants) reported not receiving counseling from healthcare providers regarding the services offered at the PNC. Sixty-five (59%) of those who were not counseled by healthcare providers about the services provided in the PNC did not attend the PNC after the last delivery. However, 22 (63%) mothers who received counseling about the services provided in the postnatal visit attended the clinic (p = 0.027).

Sixty-one (42%) participants were not informed about the booking process of the PNC. Forty-three (71%) of those who were not informed about the booking process of the PNC did not attend the PNC after the last delivery. On the other side, 50 (59%) participants of those who received counseling attended the clinic (p < 0.001) (Table 2).

**Table 2:** Association between attendance of postnatal clinic and counseling given by healthcare providers (n = 146). PNC: postnatal clinic.

Variable	Number (%)	PNC attendance, N (%)		Chi-square p-value
		No	Yes	
Explanation of the importance of postnatal clinic				0.046
No	96 (65.8)	57 (59.4)	39 (40.6)	
Yes	50 (34.2)	21 (42)	29 (58)	
Explanation of services provided in PNC				0.027
No	111 (76)	65 (58.6)	46 (41.4)	
Yes	35 (24)	13 (37.1)	22 (62.9)	
Explanation of the booking process				<0.001
No	61 (41.8)	43 (70.5)	18 (29.5)	
Yes	85 (58.2)	35 (41.2)	50 (58.8)	

There was a significant association between attendance of PNC and the mode of delivery. Attendance was higher among those who had vaginal delivery compared to C-sections. Sixty-three (32%) mothers attended the PNC among participants who had vaginal delivery while only 24 (20%) mothers attended the clinic among those who had C-sections (p < 0.03).

There was no statistically significant relationship between attendance and age, level of education and employment status of the mother, husband's level of education, number of deliveries, intention of pregnancy, and attendance of antenatal care clinic in the last pregnancy.

**Figure 3:**
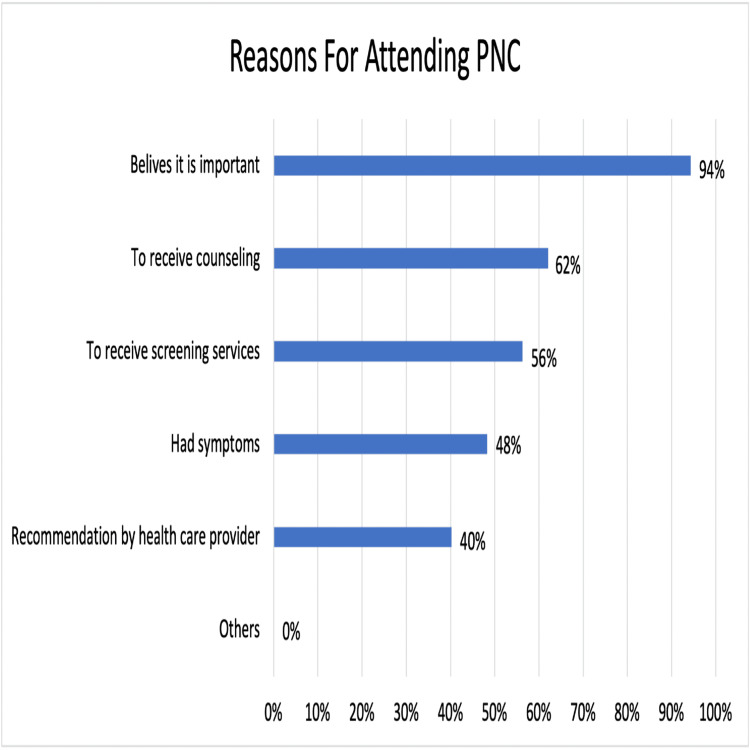
Respondents’ reasons for attending PNC (n = 87; multi-response question). PNC: postnatal clinic.

**Figure 4:**
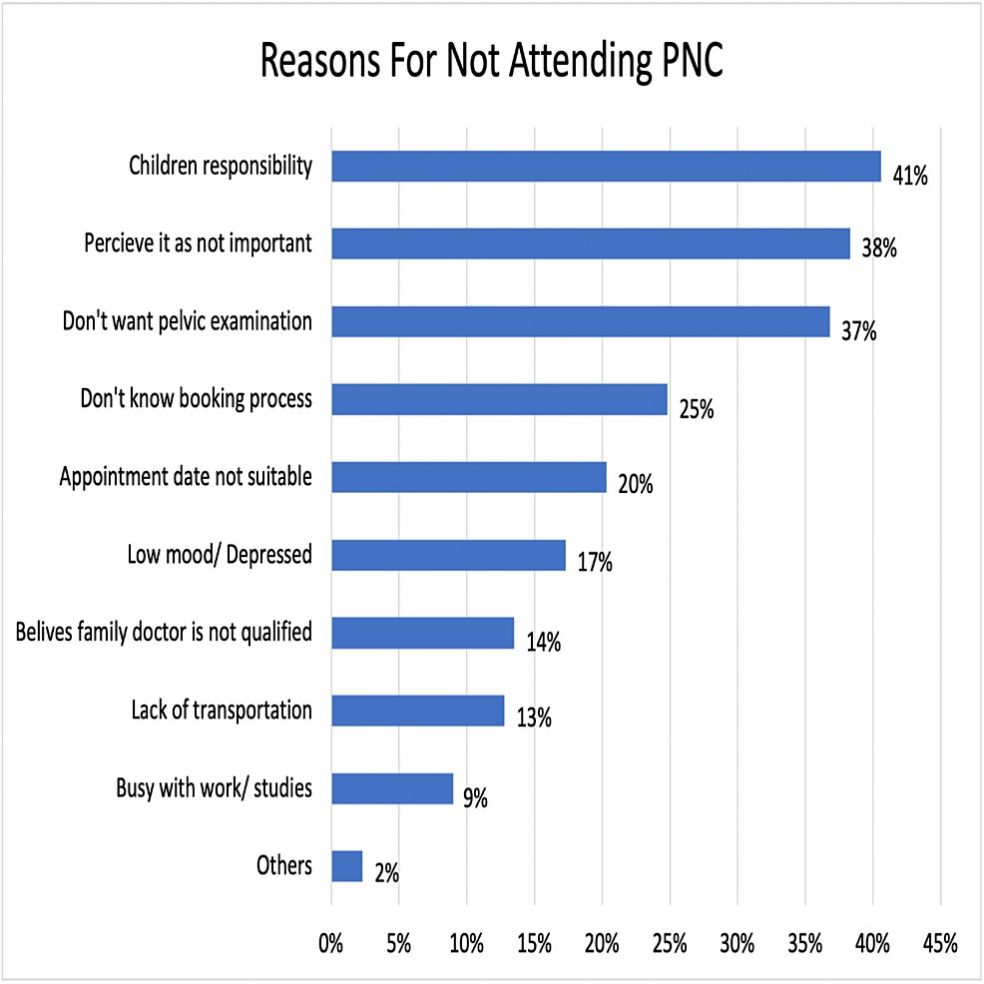
Respondents’ reasons for not attending PNC (n = 133; multi-response question). PNC: postnatal clinic.

A multi-response question was employed to inquire with 87 participants who had visited the PNC after their most recent delivery about their motivations for attending (Figure 3). Similarly, a multi-response question was used to survey 133 mothers who were aware of the PNC's existence but had not attended, seeking their reasons for not doing so (Figure 4).

Participants who had more than one delivery were asked about previous experience(s) of attending PNC after their previous deliveries (excluding the most recent one) (N = 162), to study the relation between having a previous experience(s) of attending PNC and the attendance after the last delivery. Eighty-seven (54%) of them have previous experience of attending the PNC, as opposed to 75 (46%) who do not.

There was a significant association between attendance of PNC after the last delivery and previous PNC attendance. Attendance of PNCs was higher among those who attended PNCs previously. Forty-six (53%) participants who attended PNCs previously attended the PNC after the last delivery as well. On the other side, 62 (83%) participants who did not attend a PNC(s) previously did not attend the PNC after the last delivery (p < 0.001).

There was no statistically significant association between attendance of PNC after the last delivery and long waiting time before the consultation in the previous PNC visit(s). Also, there was no statistically significant association between attendance and mother’s satisfaction with healthcare providers (doctors/nurses) in the previous PNC visits.

## Discussion

PNCs in Bahrain provide many important services to mothers. It was found that only 27% [28] of new mothers attended PNCs. This is almost similar to the proportion reported in 2019, which was 29% [28]. Similar attendance proportions were reported in the Hawassa Zuria district [28].

This study showed no significant association between PNC attendance and the age of the mother, in contrast to what was found in studies in Hawassa Zuria, which showed that the use of PNC services was higher in those under the age of 25 years [28], and in Sub-Saharan Africa, where underutilization of PNC services was high among the young age group [31]. Also, there was no significant association between PNC attendance and the educational level of both partners and the employment status of the mother, unlike what was found in Ethiopia [33], Malawi [36], Nepal [37], and Nigeria [38], where mothers who attended college or had higher education were more likely to use PNC services compared to women with primary education or no formal education. Moreover, employed women had higher PNC service attendance than the unemployed in Sub-Saharan Africa [31], Abia State [35], and Nigeria [38]. The differences in results between this study and previous research can be attributed to variations in demographic characteristics, healthcare systems, cultural norms, and socioeconomic factors across different regions and populations. Factors such as access to healthcare facilities, awareness of postnatal care services, availability of resources, and societal attitudes toward maternal and child health may influence the utilization of PNC services differently in various settings.

This study showed that women who had a cesarean delivery were more likely not to attend the PNC compared to those who had a vaginal delivery (p < 0.03), which is contradictory to the results of a study conducted in southwestern Nigeria that showed individuals who gave birth through a cesarean section were five times more likely to visit PNCs [30]. This could be explained by the mothers' misconceptions in our community about the PNCs, believing it only involves pelvic examination and is only for women who deliver vaginally. This is an issue of lack of awareness. Other obstetric histories, including the number of deliveries, intention of pregnancy, acceptance of an unplanned pregnancy, and attendance at antenatal clinic visits, were not significantly associated with PNC attendance in this study. The association between early postnatal care service utilization and factors indicative of planned and supported pregnancy, as indicated in the study from Hawassa Zuria district [28], contrasts with the findings of our study. Also, studies conducted in Sub-Saharan Africa [31], Ethiopia [33,39], and Nigeria [38] found that receiving antenatal care was positively correlated with PNC service utilization.

This study showed that there is a significant statistical association between attendance at the PNC and the information provided by healthcare workers about the importance of the PNC (p < 0.05), the services provided in the clinic (p < 0.03), and the booking process (p < 0.001). These findings are similar to those found in studies conducted in Malawi [34,36], Zaria-Nigeria [38], and Nepal [43].

This study also found a positive association between previous attendance at the PNC and attending the PNC after the last delivery (p < 0.001). This could be due to the fact that mothers who attended the PNC previously are more aware of the importance and services provided in the clinic.

In this study, 31% of the participants were unaware of the presence of PNCs in PHC centers in Bahrain, which is the reason that contributed to low attendance at the PNC. Of those who are aware, only 16% and 11% have selected “Doctor during antenatal visits” and “Nurse/midwife during antenatal visits,” respectively, as sources for their knowledge about the PNC. Mother and child health (MCH) department clerks and health educational boards in PHC centers were selected as sources of knowledge by 34% and 16% of participants, respectively. In contrast, “family member” was the most highly selected source of knowledge among participants (61%). This raises the issue that the PHC system should increase the awareness level of PNCs among mothers through doctors and nurses/midwives during antenatal visits, MCH department clerks, and health educational boards.

Among participants who selected a healthcare provider as their source of knowledge (n = 146), only 34% received counseling about the importance of the PNC, and 24% received counseling about the services provided in the PNC. A higher percentage of them (58%) received information about the booking process. These findings were significantly associated with the attendance of PNCs, which means that spending time explaining the importance, services provided, and booking process of the PNC by healthcare providers will increase the attendance rate.

Among those who have previous experience attending PNCs (excluding attendance after the most recent delivery) (n = 87), only 20% waited too long before starting the consultation. A total of 89% and 90% were satisfied with the way the doctor and nurse treated them, respectively. Unlike the results of a study in Nakasongola district [41], these findings were not significantly associated with attending the PNC, which means that waiting time and patient satisfaction with healthcare providers are not contributors to the low PNC attendance in Bahrain.

This study also supports the results from previous studies [28,34,36,38] in that most women (94%) who attended the PNC did so because they thought it was important for their health and to take advantage of the clinic's counseling and screening services. Only 40% reported attending because of their healthcare providers' encouragement. This finding is important as it suggests that mothers should receive more consistent and adequate motivation to attend the PNC. When mothers were asked about the reasons they did not seek PNC, 41% of the participants selected “lack of social support and children's responsibility” as a reason, and this was the most highly selected reason among the others. Moreover, our findings align with past research from Nepal [43], which confirmed that a lack of awareness about the importance of postnatal services was a reason for many mothers (38%) not attending PNC. Although mothers have the right to refuse it, 37% of the participants did not attend PNC because of their fear of having a pelvic examination. This can be easily avoided if healthcare professionals adequately explain the services offered at the postnatal visit. Because 42% of women did not receive any explanation about the booking process for the PNC from their healthcare provider, it is not surprising that 25% of women did not attend because they were uncertain about how to book for it. Unlike one Indian study [42], our findings suggest that low utilization of these services is not mainly due to a lack of trust in family physicians, as only 14% of participants selected this option, and this indicates a good doctor-patient relationship. Furthermore, unlike earlier studies done in Nigeria [38] and Zambia [40], we did not identify transportation as a significant factor in this study. This can be justified by the fact that the PHC centers in Bahrain are distributed fairly among the different populated areas, and the furthest house from any center is around 10 minutes by car, and almost all families have cars.

Attempts to improve postnatal care utilization in Bahrain should concentrate on raising the community's awareness about the significant role of PNCs and the services they provide among expectant mothers. The right counseling encounters during antenatal appointments are very crucial and this can correct the existing myths of pregnancy and encourage attendance. Community engagement particularly in the form of family involvement is considered absolutely crucial to surmounting socio-cultural barriers. It is important to enroll PHC facilities and grow patient-oriented services to strengthen patients' contentment and develop patient-provider links. Enhancing postnatal continuity of care for multiple deliveries and strengthening across health systems are some of the main strategies for the improvement of the mothers' health outcomes. By adopting these actions, stakeholders can collaborate to improve the use of postnatal services and ultimately enhance maternal and child health in Bahrain.

The data for this study came from a large and nationally representative survey, as it was gathered from all the medical regions in Bahrain. Moreover, this study used an interview as a data collection tool, which minimized the misunderstanding of the questions by the participants and reduced the errors that could happen with the skip patterns in the questionnaire. Standardized techniques that may be applied in many countries were used.

This study has some limitations. The cross-sectional design of the study and the reporting of previous experience may have resulted in recall bias. Despite these limitations, the findings of this study close a significant gap in the regional literature and offer key information for the promotion of maternal health in Bahrain.

## Conclusions

PNC attendance among mothers in Bahrain faces challenges, with 31% unaware of the presence of this clinic in the PHC centers. A limited number received information from healthcare providers, highlighting the need for enhanced antenatal counseling. Emphasizing the significance of postnatal visits, explaining services, and streamlining the booking process could notably boost attendance, addressing key factors contributing to non-attendance.

To enhance awareness, we propose incorporating postnatal visits into antenatal counseling during the third trimester and introducing online booking and rescheduling services. Considering varied schedules, offering evening hours, and tele-consultations accommodate mothers with childcare responsibilities. Furthermore, optimizing health education tools, such as boards, brochures, short message service (SMS), and media, alongside strengthening department clerks' roles, will effectively disseminate information and emphasize the importance of postnatal care.
